# A multicenter comparison of [^18^F]flortaucipir, [^18^F]RO948, and [^18^F]MK6240 tau PET tracers to detect a common target ROI for differential diagnosis

**DOI:** 10.1007/s00259-021-05401-4

**Published:** 2021-05-27

**Authors:** Antoine Leuzy, Tharick A. Pascoal, Olof Strandberg, Philip Insel, Ruben Smith, Niklas Mattsson-Carlgren, Andréa L. Benedet, Hannah Cho, Chul H. Lyoo, Renaud La Joie, Gil D. Rabinovici, Rik Ossenkoppele, Pedro Rosa-Neto, Oskar Hansson

**Affiliations:** 1 Clinical Memory Research Unit, Department of Clinical Sciences, Lund University, Malmö, Sweden; 2 Translational Neuroimaging Laboratory, McGill Centre for Studies in Aging, McGill University, Montreal, QC Canada; 3 Department of Psychiatry and Neurology, University of Pittsburgh, Pittsburgh, PA USA; 4 Department of Psychiatry, University of California, San Francisco, CA USA; 5 Department of Neurology, Skåne University Hospital, Lund, Sweden; 6 Wallenberg Centre for Molecular Medicine, Lund University, Lund, Sweden; 7 CAPES Foundation, Ministry of Education of Brazil, Brasília, Brazil; 8 Department of Neurology, Gangnam Severance Hospital, Yonsei University College of Medicine, Seoul, South Korea; 9 Memory and Aging Center, Department of Neurology, Weill Institute for Neurosciences, University of California, San Francisco, San Francisco, CA USA; 10 Departments of Neurology, Radiology & Biomedical Imaging, University of California San Francisco, San Francisco, USA; 11 VU University Medical Center, Neuroscience Campus Amsterdam, Amsterdam, The Netherlands; 12 Montreal Neurological Institute, Montreal, QC Canada; 13 Department of Neurology and Neurosurgery, McGill University, Montreal, QC Canada; 14 Memory Clinic, Skåne University Hospital, Lund, Sweden

**Keywords:** Tau, PET, [^18^F]Flortaucipir, [^18^F]RO948, [^18^F]MK6240

## Abstract

**Purpose:**

This study aims to determine whether comparable target regions of interest (ROIs) and cut-offs can be used across [^18^F]flortaucipir, [^18^F]RO948, and [^18^F]MK6240 tau positron emission tomography (PET) tracers for differential diagnosis of Alzheimer’s disease (AD) dementia vs either cognitively unimpaired (CU) individuals or non-AD neurodegenerative diseases.

**Methods:**

A total of 1755 participants underwent tau PET using either [^18^F]flortaucipir (*n* = 975), [^18^F]RO948 (*n* = 493), or [^18^F]MK6240 (*n* = 287). SUVR values were calculated across four theory-driven ROIs and several tracer-specific data-driven (hierarchical clustering) regions of interest (ROIs). Diagnostic performance and cut-offs for ROIs were determined using receiver operating characteristic analyses and the Youden index, respectively.

**Results:**

Comparable diagnostic performance (area under the receiver operating characteristic curve [AUC]) was observed between theory- and data-driven ROIs. The theory-defined temporal meta-ROI generally performed very well for all three tracers (AUCs: 0.926–0.996). An SUVR value of approximately 1.35 was a common threshold when using this ROI.

**Conclusion:**

The temporal meta-ROI can be used for differential diagnosis of dementia patients with [^18^F]flortaucipir, [^18^F]RO948, and [^18^F]MK6240 tau PET with high accuracy, and that using very similar cut-offs of around 1.35 SUVR. This ROI/SUVR cut-off can also be applied across tracers to define tau positivity.

**Supplementary Information:**

The online version contains supplementary material available at 10.1007/s00259-021-05401-4.

## Introduction

In addition to neuritic plaques composed of amyloid-β (Aβ), Alzheimer’s disease (AD) is characterized by tau pathology, largely in the form of paired helical filaments (PHFs) comprising a mixture of three/four-repeat tau isoforms [1]. While tau aggregates are also present in various non-AD neurodegenerative disorders, including certain variants of frontotemporal lobar degeneration and progressive supranuclear palsy (PSP), these are structurally distinct from those observed in AD [2, 3].

The development of radiotracers selective for tau aggregates for use with positron emission tomography (PET) has allowed for their visualization and quantification in vivo [4]. The most widely used tau tracer to date, [^18^F]flortaucipir [5], has been shown to primarily detect AD-type tau aggregates [6, 7]; as such, tau PET may be most valuable for differentiating AD from non-AD tauopathies and other neurodegenerative disorders. Though tau PET is a relatively recent technique, several novel tracers characterized by improved specificity and dynamic range have recently entered the field, including [^18^F]RO948 [8] and [^18^F]MK6240 [9, 10]. While characterized by improved specificity and dynamic range, these tracers also show greater meningeal uptake.

Several approaches have been proposed to quantify regional tau pathology. While some have applied theory-driven regions of interest (ROIs) based on *post-mortem* findings (i.e., approximating the Braak staging scheme [11–13] for tau pathology), others have used approaches that are driven solely by the spatial patterns contained in tau PET images (i.e., data driven) [14–17]. As post-mortem and in vivo PET studies have shown that tau deposition patterns can deviate significantly from the Braak staging scheme [13, 18–23], data-driven approaches may provide a more accurate measure of tau burden. It is unclear, however, which of the two approaches (i.e., theory- or data-driven) is optimal for diagnostic purposes.

With an increasing number of available tau tracers, there will also be greater variability in quantitative outcome measures. This variability is due to the distinct properties of each tau tracer, and to different acquisition procedures across sites, analytical methods, and ROI selection. In contrast to amyloid PET, however, where outcome measures from different tracers or methods can be standardized to a common scale [24], tau PET findings cannot currently be directly comparable. A common scale for tau imaging would facilitate direct comparison of outcome measures and tracer characteristics, and help establish uniform cut-offs for early tau pathology and the range of tau positivity characteristic of AD.

Using [^18^F]flortaucipir, [^18^F]RO948, and [^18^F]MK6240, the objectives of the present study were to compare the diagnostic performance (i.e., separating AD dementia from cognitively unimpaired (CU) individuals and non-AD disorders) of theory- and data-driven ROIs in order to examine whether a common target ROI (and cut-off to define tau PET positivity) can be used across tracers for differential diagnosis. In addition, at a broader level, comparison of findings across these three tracers can serve as a proof of concept with respect to the eventual standardization of tau PET imaging measures.

## Materials and methods

### Participants

A total of 1755 participants were included from seven different cohorts. [^18^F]Flortaucipir data was drawn from a convenience sample of participants recruited from the Memory Disorder Clinic of Gangnam Severance Hospital (Seoul, South Korea), the Swedish BioFINDER study (clinical trial no. NCT01208675) at Lund University (Lund, Sweden), and the University of California San Francisco Alzheimer Disease Research Center (UCSF, USA). Additional [^18^F]flortaucipir scans were collected from the Avid Radiopharmaceuticals Study A05e (NCT 02016560) and the placebo arm of the Expedition-3 study. For [^18^F]RO948, subjects were drawn from the prospective and longitudinal Swedish BioFINDER-2 study (clinical trial no. NCT03174938). [^18^F]MK6240 PET data was obtained from the prospective and longitudinal TRIAD (Translational Biomarkers in Aging and Dementia) cohort at McGill University (Montreal, Canada). Groups were established without the use of biomarkers. We included only patients with Aβ-positive AD dementia in accordance with the National Institute on Aging-Alzheimer Association research framework [25]. Additional details on included cohorts and on the definition of study groups are included in Supplementary Tables 1–4. Informed consent was obtained from all participants, with studies approved by local institutional review boards.

### Image acquisition and processing

Complete details on the acquisition and processing of tau PET data have been described elsewhere [9, 11, 26–28]. Briefly, [^18^F]flortaucipir PET data was acquired over the post-injection interval of 80–100 min; [^18^F]RO948 and [^18^F]MK6240 data were acquired 70–90 and 90–110 min after injection, respectively. All participants also had an anatomical 3D T1-weighted magnetic resonance imaging (MRI) scan. [^18^F]Flortaucipir and [^18^F]RO948 images were centrally processed at Lund University while [^18^F]MK6240 data was processed at McGill University using a similar analytical pipeline. For all three tracers, images were first motion corrected, time-averaged, and rigidly coregistered to their corresponding skull-stripped T1-weighted structural MRI scan. Standardized uptake value ratio (SUVR) images were created using the inferior cerebellar cortex as reference region for all tracers. FreeSurfer (v.6.0)-based parcellations of T1-weighted MRI scans were applied to the tau PET scans transformed to participants’ native T1-space to extract mean regional SUVR values for each participant. For voxelwise analyses, SUVR PET images were spatially transformed into a common MNI152 space using the transformation derived from MRI normalization step.

### Region-of-interest definition

#### Data-driven ROIs

Ranked feature importance of ROIs was obtained for [^18^F]flortaucipir, [^18^F]RO948, and [^18^F]MK6240 through a machine learning algorithm called Extra Trees (ETs) [29]. In short, ETs are similar to the better-known Random Forest. As ETs fit each tree on all data without bagging, calculations were evaluated in 10-fold cross-validation loops yielding mean feature importance and standard deviations. Subjects of two subgroups (first AD dementia and CU, followed by AD dementia and non-AD) and their feature vectors (mean SUVR values in FreeSurfer ROIs) were used to train the ETs to predict group membership by decision tree majority vote and output the feature importance scores extracted from the ensemble. The highest ranked features from each resulting group were then extracted from the resulting dendrogram, first out of a single group (all features) and subsequently from the highest ranked groups in an iterative fashion. This allowed us to assess the relationship between accuracy and the number of group representatives added, providing a truly minimalist representation of group differentiating ROIs. The dendrograms resulting from this approach are provided for each tracer and contrast in Supplementary Fig. 1–6.

#### Theory-driven ROIs

In order to include brain areas affected by NFT pathology across the course of AD (i.e., from early to later affected areas), we created four FreeSurfer-based composite ROIs using an approach developed using [^18^F]flortaucipir [11] and based on the Braak staging scheme for tau pathology [30]. These ROIs have been used previously [31, 32] and include the entorhinal cortex (stage I/II), a temporal meta-ROI (entorhinal cortex, amygdala, inferior/middle temporal gyri, fusiform gyrus, and parahippocampal gyrus, approximating Braak I/IV) [33, 34], and a neocortical meta-ROI (widespread neocortical areas, approximating Braak V/VI). In addition, we included an Early tau ROI comprising regions shown to accumulate tau early on in the course of AD (entorhinal cortex, inferior temporal cortex, fusiform gyrus, and parahippocampal gyrus) [35]. Despite substantial anatomical overlap with the temporal meta-ROI, the inclusion of this Early tau ROI was motivated by findings showing that tau PET signals in these different temporal regions follow different dynamics when it comes to tau accumulation, indicating that small differences in ROI composition may affect sensitivity [36].

### Statistical analyses

All analyses were performed in R, v.4.0.2 (R Foundation for Statistical Computing, https:/d/www.R-project.org/), with significance set at *P* < 0.05, two-tailed. Demographics and tau PET SUVR values at the ROI level were compared across cohorts and diagnostic groups using analysis of variance and post hoc t-tests (continuous variables) or Fisher’s exact tests (binary variables). Cut-offs for tau PET imaging ROIs were determined for each tracer using the Youden index (sensivity+specificity-1; AD dementia vs Aβ-negative CU). The diagnostic performance of tau PET (AD dementia vs CU and non-AD) was assessed for each ROI by testing for significant differences between area under the receiver operating characteristic curve (AUC) values for ROIs using DeLong statistics [37].

## Results

### Participant characteristics

**Fig. 1:**
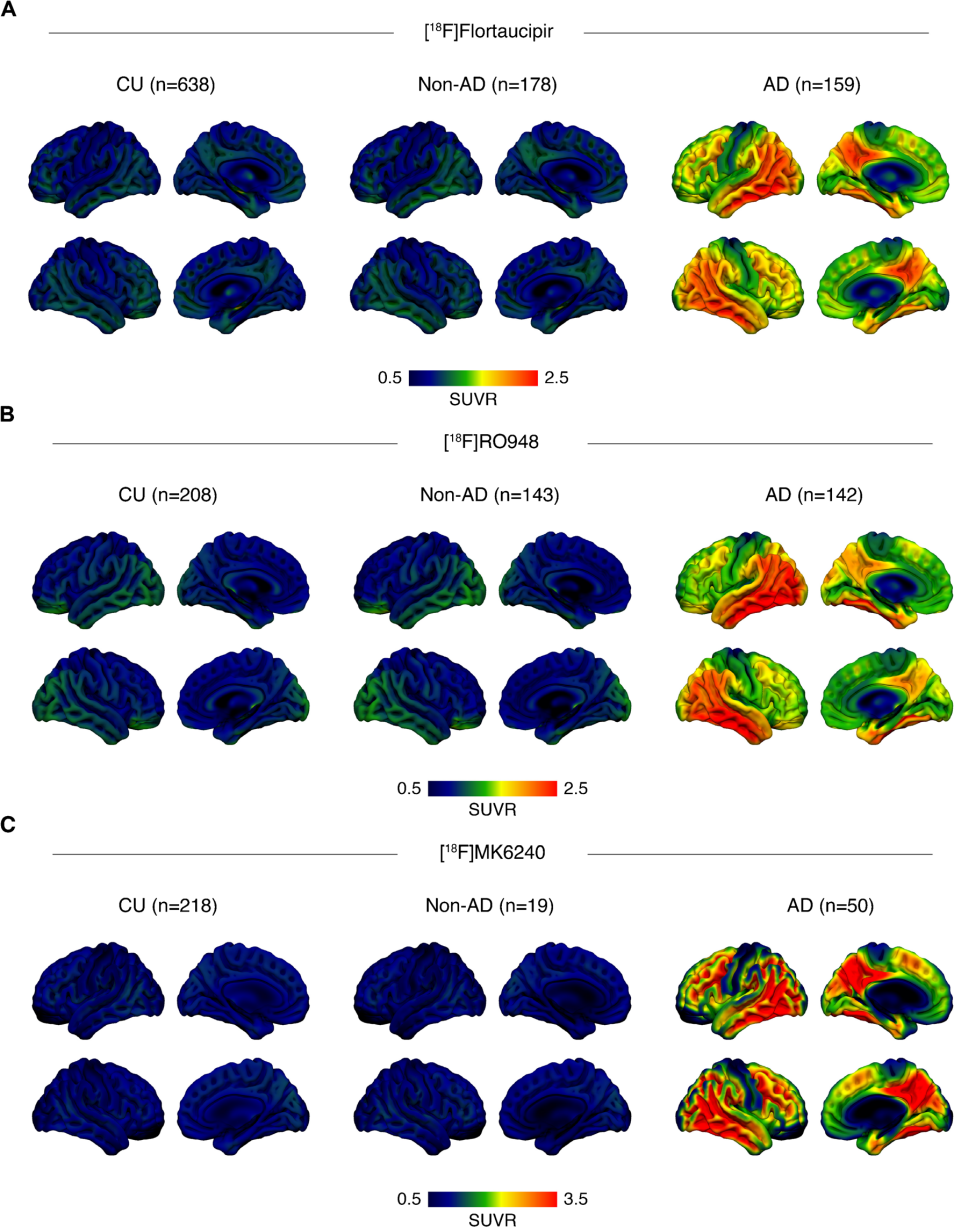
Mean [^18^F]flortaucipir (**A**), [^18^F]RO948 (**B**), and [^18^F]MK6240 (**C**) standardized uptake values ratios (SUVRs) across all participants within diagnostic groups

For [^18^F]flortaucipir, we included 975 subjects, including 638 CU individuals, 178 non-AD disorders (15 corticobasal syndrome (CBS), 18 dementia with Lewy bodies (DLB), three multiple system atrophy (MSA), 65 Parkinson’s disease with or without cognitive impairment (PD/PDD), 14 progressive non-fluent aphasia (PNFA), six with semantic dementia (SD), 26 with behavioral variant frontotemporal dementia (bvFTD), four with vascular dementia (VaD), and 27 progressive supranuclear palsy (PSP)), and 159 patients with AD dementia. For [^18^F]RO948, we included a total of 493 subjects (208 CU, 143 non-AD (three CBS, 30 DLB, 23 FTD, 13 MSA, 47 PD/PDD, three PNFA, and 24 PSP), and 142 AD dementia) while for [^18^F]MK6240 we included a total of 287 subjects (218 CU, 19 non-AD (one CBS, 14 FTD, one PPA, and three PSP), and 50 AD dementia). Participant characteristics are summarized in Table 1 with average tau PET images across diagnostic groups and tracers shown in Fig. 1.

**Table 1:** Cohort characteristics

	[^18^F]Flortaucipir			[^18^F]RO948			[^18^F]MK6240		
	CU	Non-AD	AD	CU	Non-AD	AD	CU	Non-AD	AD
*N*, %F	638 (57%)	178 (45%)	159 (60%)	208 (56%)	143 (41%)	142 (54%)	218 (66%)	19 (59%)	50 (56%)
Age, yr	69.74 (9.03)	68.16 (8.15)	70.72 (10.38)	75.29 (5.94)	70.17 (9.15)	74.14 (6.68)	61.56 (19.78)	62.52 (8.88)	66.10 (10.01)
Education, yr	14.70 (4.60)	13.22 (5.71)	11.87 (6.20)	12.01 (3.72)	12.54 (3.64)	12.20 (4.40)	15.72 (3.54)	13.95 (4.20)	14.00 (3.93)
MMSE	28.96 (1.27)	23.28 (6.21)	20.40 (5.08)	28.78 (1.23)	25.27 (4.90)	20.35 (4.12)	29.22 (1.09)	24.88 (7.06)	18.49 (5.97)
APOE ε4 carrier, *n* (%)^*^	107 (27%)	32 (28%)	76 (59%)	82 (42%)	53 (39%)	102 (72%)	58 (28%)	2 (12%)	24 (55%)
Aβ positive, *n* (%)^**^	226 (35%)	29 (19%)	159 (100%)	90 (43%)	52 (34%)	142 (100%)	47 (22%)	0 (0%)	50 (100%)
SUVR, theory-driven (apriori) ROIs									
Entorhinal cortex	1.14 (0.12)	1.22 (0.21)	1.74 (0.32)	1.23 (0.22)	1.22 (0.31)	2.00 (0.40)	0.93 (0.23)	0.84 (0.21)	2.42 (0.56)
Early tau	1.19 (0.11)	1.22 (0.19)	1.88 (0.46)	1.23 (0.18)	1.22 (0.24)	2.15 (0.66)	0.87 (0.12)	0.81 (0.09)	2.80 (0.64)
Temporal meta-ROI	1.18 (0.11)	1.20 (0.16)	1.88 (0.47)	1.22 (0.18)	1.22 (0.23)	2.13 (0.66)	0.86 (0.11)	0.82 (0.09)	2.84 (0.66)
Neocortical meta-ROI	1.08 (0.08)	1.13 (0.15)	1.53 (0.38)	1.23 (0.18)	1.08 (0.16)	1.51 (0.42)	1.02 (0.10)	0.98 (0.10)	2.81 (0.97)

^*^Missing *APOE* data: [^18^F]flortaucipir (237 CU, 64 non-AD, 32 AD); [^18^F]RO948 (3 non-AD); [^18^F]MK6240 (13 CU, 2 non-AD, 6 AD); ^**^Aβ-status was missing in 31 non-AD subjects for [^18^F]flortaucipir

**Fig. 2:**
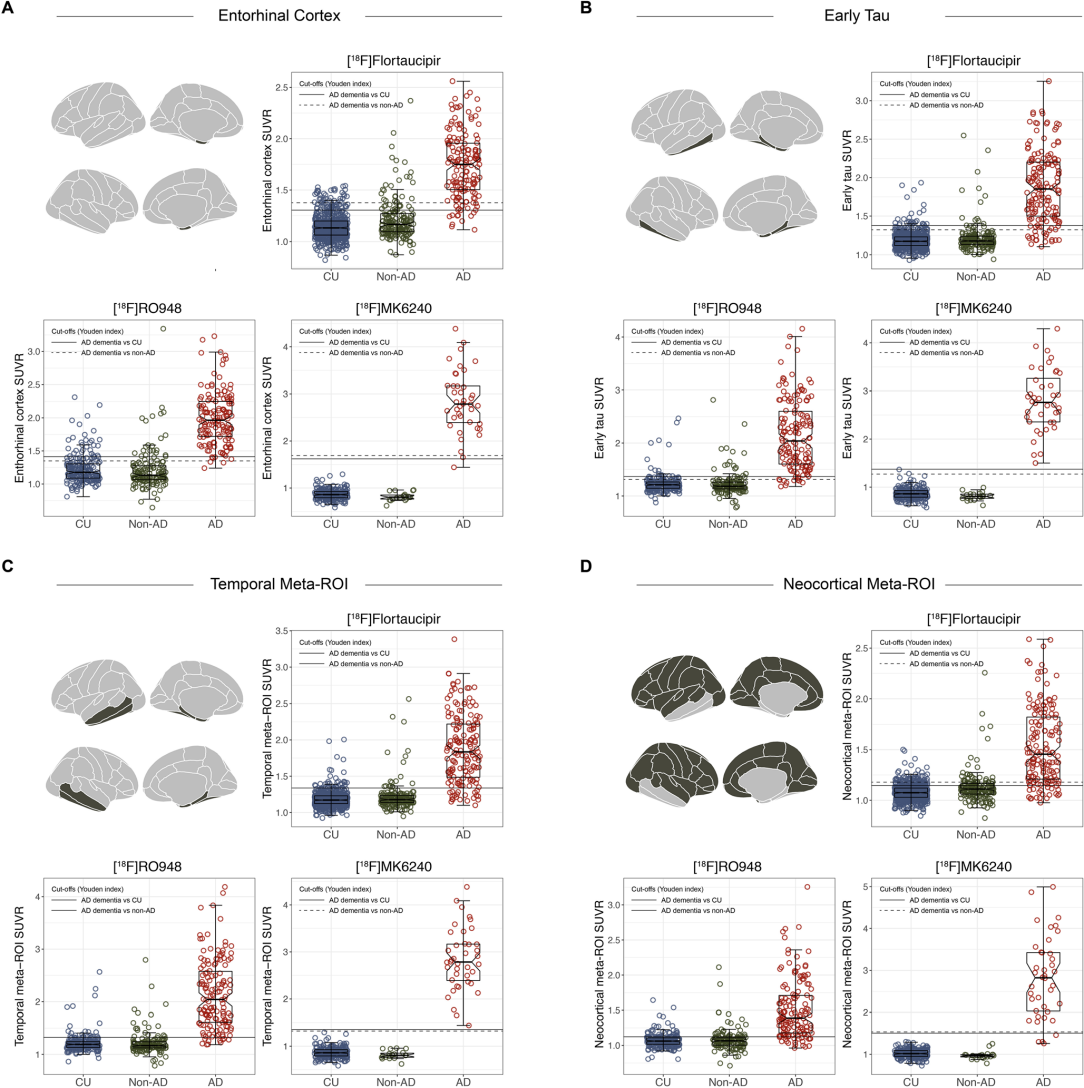
Distribution of SUVR values for [^18^F]flortaucipir, [^18^F]RO948, and [^18^F]MK6240 across theory-driven ROIs. (**A**) Entorhinal cortex; (**B**) Early tau; (**C**) temporal meta-ROI; (**D**) neocortical meta-ROI. In each panel, the upper left figure is a representation of the ROI used (i.e., individual FreeSurfer-based regions, displayed on left and right hemispheres); the remaining plots show SUVR values for each tracer across diagnostic groups. The notch in the box-and-whisker plots indicates the 95% confidence interval for the median

SUVR values across tracers and diagnostic groups are shown for theory-driven ROIs in Fig. 2. For all three tracers, SUVR values across apriori ROIs were significantly higher in AD patients as compared to CU individuals and non-AD disorders (*P* < 0.001). For [^18^F]flortaucipir, SUVR values in the non-AD group were significantly higher than those for CU individuals in the entorhinal cortex (*P* < 0.001), in the Early tau ROI (*P* < 0.05) and in the neocortical meta-ROI (*P* < 0.001). For [^18^F]MK6240, SUVR values were significantly higher in CU individuals compared to non-AD in the Early tau and temporal meta-ROIs (Table 1).

### Deriving data-driven ROIs

**Fig. 3:**
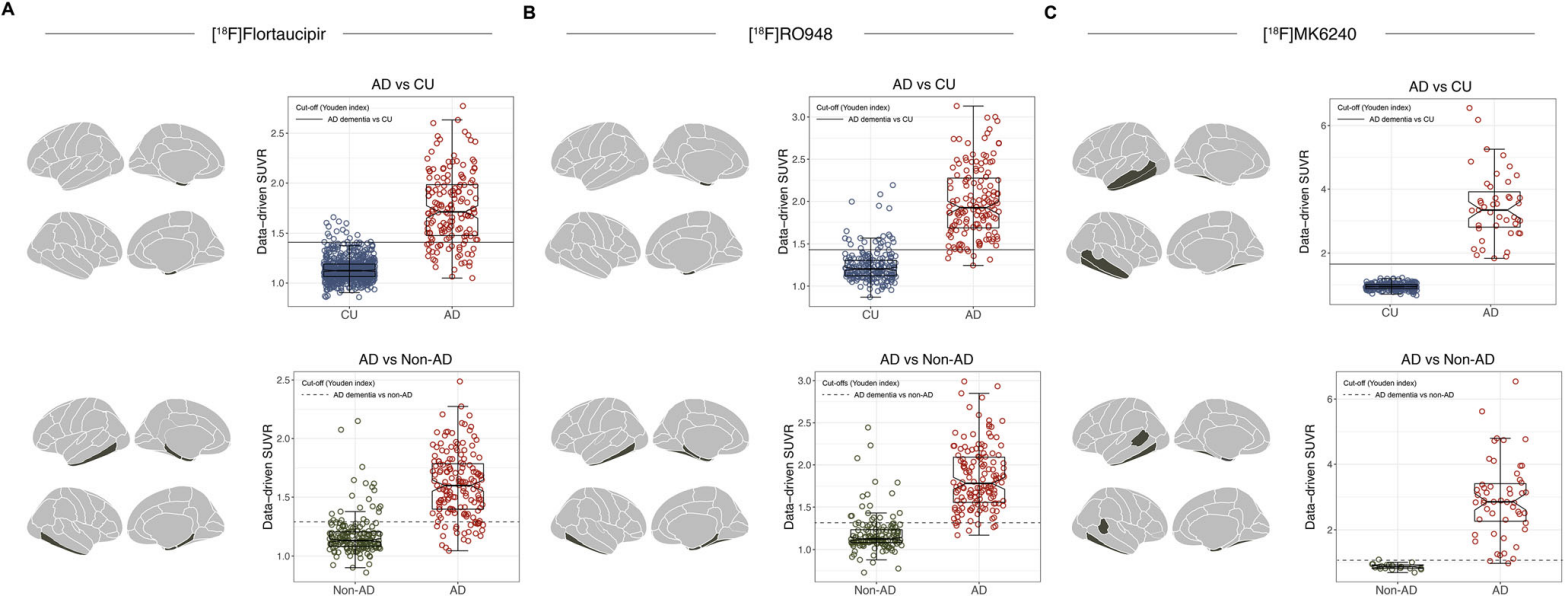
Distribution of SUVR values for [^18^F]flortaucipir, [^18^F]RO948, and [^18^F]MK6240 across data-driven ROIs. The notch in the box-and-whisker plots indicates the 95% confidence interval for the median. (**A**) The regions that best separated AD dementia from CU individuals (parahippocampus and inferior temporal cortex) and those diagnosed with non-AD neurodegenerative disorders (entorhinal cortex, amygdala, parahippocampus, and inferior temporal cortex) using [^18^F]flortaucipir PET. (**B**) The regions that best separated AD dementia from CU individuals (entorhinal cortex and amygdala) and those diagnosed with non-AD neurodegenerative disorders (entorhinal cortex, amygdala, parahippocampus, fusiform gyrus, and inferior temporal cortex) using [^18^F]RO948. (**C**) The regions that best separated AD dementia from CU individuals (fusiform gyrus, inferior temporal cortex, middle temporal gyrus) and those diagnosed with non-AD neurodegenerative disorders (entorhinal cortex, amygdala, the inferior temporal cortex, the banks of the superior temporal sulcus, and the fusiform gyrus) using [^18^F]MK6240

Extra-tree-based data-driven ROIs are shown by tau tracer in Fig. 3. For separating AD dementia from CU individuals (A), data-driven ROIs encompassed the following regions: for [^18^F]flortaucipir, the inferior temporal cortex and parahippocampal gyrus (AUC 0.966, 95% CI 0.949–0.983); for [^18^F]RO948, the entorhinal cortex and the amygdala (AUC 0.969 95% CI 0.953–0.984); for [^18^F]MK6240, the inferior temporal cortex, the fusiform gyrus, and the middle temporal cortex (AUC 0.988, 95% CI 0.974–1). For separating AD dementia from non-AD disorders (B), data-driven ROIs encompassed the entorhinal cortex, the amygdala, the parahippocampus, and the inferior temporal cortex for [^18^F]flortaucipir (AUC 0.926, 95% CI 0.895–0.956); the entorhinal cortex, parahippocampus, amygdala, fusiform gyrus, and inferior temporal cortex for [^18^F]RO948 (AUC 0.956, 95% CI 0.931–0.981); and the entorhinal cortex, amygdala, the inferior temporal cortex, the banks of the superior temporal sulcus, and the fusiform gyrus for [^18^F]MK6240 (AUC 0.997, 95% CI 0.991–1).

### Establishing cut-offs for data- and theory-driven ROIs

Cut-offs for SUVR values within tau PET imaging ROIs (data- and theory-driven) were determined across tracers and are presented in Table 2. For the separation of AD dementia from non-AD disorders, the temporal meta-ROI showed the highest AUC for all three tracers; moreover, cut-off values appeared to converge around an SUVR of 1.35 (average value; [^18^F]flortaucipir, 1.36 (95% CI, 1.31, 1.46); [^18^F]RO948, 1.34 (95% CI, 1.27, 1.43); [^18^F]MK6240, 1.34 (95% CI, 1.15, 1.42)).

**Table 2:** Summary of diagnostic performance and cut-offs for [^18^F]flortaucipir, [^18^F]RO948, and [^18^F]MK6240. *AUC*, area under the receiver operating curve value; *95% CI*, 95% confidence interval

	AUC (95% CI)	Cut-point (95% CI)	Sensitivity (95% CI)	Specificity (95% CI)
[^18^F]Flortaucipir				
AD vs CU				
Entorhinal cortex	0.959 (0.941–0.977)	1.30 (1.18, 1.35)	92.30 (88.10–96.20)	87.80 (85.10–90.30)
Early tau	0.941 (0.917–0.965)	1.35 (1.28, 1.42)	86.20 (80.10–91.20)	94.40 (92.30–95.90)
Temporal meta-ROI	0.942 (0.917–0.965)	1.36 (1.34, 1.40)	86.20 (79.90–90.60)	95.10 (93.40–96.70)
Neocortical meta-ROI	0.901 (0.871–0.938)	1.17 (1.13, 1.21)	59.10 (50.90–66.70)	99.20 (98.60–99.80)
Data-driven^1^	0.958 (0.942–0.983)	1.33 (1.25, 1.41)	89.30 (84.30–93.70)	92.00 (89.90–94.10)
AD vs non-AD				
Entorhinal cortex	0.919 (0.889–0.949)	1.36 (1.29, 1.45)	87.40 (82.40–92.30)	85.40 (80.30–90.50)
Early tau	0.925 (0.895–0.955)	1.31 (1.21, 1.36)	88.10 (83.00–92.50)	89.90 (85.40–94.40)
Temporal meta-ROI	0.926 (0.896–0.956)	1.36 (1.31, 1.46)	84.90 (79.30–90.60)	93.30 (89.30–96.60)
Neocortical meta-ROI	0.859 (0.816–0.901)	1.19 (1.09, 1.23)	78.70 (72.30–84.90)	84.30 (78.90–89.30)
Data-driven^2^	0.925 (0.895–0.955)	1.27 (1.20, 1.29)	88.70 (83.70–93.10)	89.30 (84.80–93.40)
[^18^F]RO948				
AD vs CU				
Entorhinal cortex	0.972 (0.956–0.987)	1.44 (1.34, 1.52)	94.40 (90.10–97.90)	87.90 (83.70–92.30)
Early tau	0.981 (0.969–0.994)	1.38 (1.33, 1.43)	90.10 (84.50–95.10)	94.70 (91.40–97.60)
Temporal meta-ROI	0.982 (0.969–0.994)	1.34 (1.24, 1.39)	92.30 (88.00–96.50)	91.80 (87.90–95.20)
Neocortical meta-ROI	0.942 (0.917–0.967)	1.13 (1.08, 1.16)	88.00 (82.40–92.90)	76.00 (70.20–81.70)
Data-driven^3^	0.980 (0.967–0.993)	1.39 (1.33, 1.48)	89.40 (83.80–94.40)	94.70 (91.40–97.60)
AD vs non-AD				
Entorhinal cortex	0.954 (0.929–0.979)	1.37 (1.18, 1.45)	97.90 (95.10–100)	83.90 (77.60–89.50)
Early tau	0.955 (0.932–0.978)	1.36 (1.29, 1.45)	91.60 (86.60–95.80)	88.10 (82.50–93.00)
Temporal meta-ROI	0.955 (0.933–0.978)	1.34 (1.27, 1.43)	89.40 (84.50–94.40)	88.80 (83.20–93.70)
Neocortical meta-ROI	0.893 (0.855–0.931)	1.13 (1.07, 1.18)	85.20 (78.90–90.90)	81.80 (77.50–87.40)
Data-driven^4^	0.956 (0.933–0.980)	1.30 (1.18, 1.38)	95.70 (92.30–98.90)	86.70 (81.10–92.30)
[^18^F]MK6240				
AD vs CU				
Entorhinal cortex	0.969 (0.944–0.994)	1.58 (1.14, 2.10)	80.30 (73.20–86.60)	93.70 (89.50–97.20)
Early tau	0.985 (0.970–1)	1.39 (1.29, 1.54)	87.30 (81.70–92.90)	90.90 (86.00–95.10)
Temporal meta-ROI	0.989 (0.979–1)	1.36 (1.16, 1.49)	90.10 (84.50–94.40)	88.80 (83.20–93.70)
Neocortical meta-ROI	0.980 (0.957–1)	1.25 (1.17, 1.30)	68.30 (61.30–76.10)	93.00 (88.80–96.50)
Data-driven^5^	0.986 (0.967–1)	1.53 (1.46, 1.58)	83.80 (77.50–89.40)	90.20 (85.30–90.20)
AD vs non-AD				
Entorhinal cortex	0.987 (0.969–1)	1.61 (1.31, 2.29)	80.30 (73.90–86.60)	93.70 (89.50–97.20)
Early tau	0.995 (0.988–1)	1.31 (1.14, 1.37)	93.70 (89.40–97.20)	83.20 (76.90–88.80)
Temporal meta-ROI	0.996 (0.987–1)	1.34 (1.15, 1.42)	92.90 (88.70–96.50)	86.70 (81.10–92.30)
Neocortical meta-ROI	0.992 (0.978–1)	1.26 (1.16, 1.36)	66.20 (58.50–73.90)	93.00 (88.10–96.50)
Data-driven^6^	0.997 (0.991–1)	1.23 (1.09, 1.32)	97.90 (95.10–100)	75.50 (68.50–82.30)

^1^Inferior temporal cortex and parahippocampal gyrus; ^2^entorhinal cortex, amygdala, parahippocampus, and inferior temporal cortex; ^3^entorhinal cortex and amygdala; ^4^entorhinal cortex, parahippocampus, amygdala, fusiform gyrus, and inferior temporal cortex; ^5^inferior temporal cortex, fusiform gyrus, and middle temporal cortex; ^6^entorhinal cortex, amygdala, the inferior temporal cortex, the banks of the superior temporal sulcus, and the fusiform gyrus

### Diagnostic performance using theory- and data-driven ROIs

AUC findings—along with sensitivity and specificity estimates—for theory- and data-driven ROIs are summarized in Table 2. When using theory-driven ROIs (i.e., entorhinal cortex, Early tau, temporal, and neocortical meta-ROIs) for the separation of AD dementia from CU individuals, the entorhinal cortex ranked highest in terms of AUC for [^18^F]flortaucipir (AUC 0.959, 95% CI 0.941–0.977) while for [^18^F]RO948 and [^18^F]MK6240, the temporal meta-ROI performed best (AUC 0.982, 95% CI 0.969–0.994; 0.989, 95% CI 0.979–1, respectively). For the separation of AD dementia from non-AD disorders, the temporal meta-ROI performed best across all three tracers: [^18^F]flortaucipir (AUC 0.926, 95% CI 0.896–0.956); [^18^F]RO948 (AUC 0.982, 95% CI 0.969–0.994); [^18^F]MK6240 (AUC 0.995, 95% CI 0.987–1). For both contrasts (i.e., AD dementia vs CU individuals and AD dementia vs non-AD), DeLong statistics showed that there were no significant differences between the AUC values from best performing theory-driven ROIs and those derived from the data-driven ROIs. Similar findings were obtained for AD dementia vs non-AD when excluding PD patients without dementia (Supplementary Table 5) and when excluding PD/PDD and DLB cases that were Aβ-positive ([^18^F]flortaucipir, *n* = 26; [^18^F]RO948, *n* = 31) (Supplementary Table 6).

## Discussion

In this multicentric study, we compared the cross-sectional diagnostic performance of [^18^F]flortaucipir, [^18^F]RO948, and [^18^F]MK6240 tau PET for the separation of AD dementia from both CU individuals and non-AD disorders, using SUVR values drawn from both theory-driven (apriori) and data-driven ROIs. For these comparisons, no significant differences in AUC values were seen between the best performing theory-driven ROIs and those determined using the data-driven approach. Moreover, an SUVR value of approximately 1.35 appeared to be a common threshold across tracers for the temporal meta-ROI (corresponding to Braak I/IV).

Ongoing work applying a functional connectivity-based approaches to define ROIs for tau PET suggests that data-driven approaches may better capture tau pathology cross-sectionally and can provide patient-tailored ROIs that predict longitudinal tau accumulation with greater sensitivity than Braak-based stages [38]. Our findings suggest, however, that the temporal meta-ROI (approximating Braak stage I/IV) is suitable for achieving high diagnostic accuracy across tau PET tracers in differentiating AD dementia from CU individuals and non-AD dementia disorders. The ranking of the temporal meta-ROI as the best performing among apriori ROI is consistent with earlier studies using both [^18^F]flortaucipir [31] and [^18^F]RO948 [32]. Recent work using [^18^F]flortaucipir has also highlighted the likely need for quantification as an adjunct to visual assessment if tau PET is to be used clinically [39]. In that study, physicians visually assessed [^18^F]flortaucipir PET images as consistent or not consistent with AD. These ratings were then compared to tau immunohistochemistry and levels of AD neuropathologic change based on Aβ plaque burden [39]. Prespecified levels of sensitivity and specificity were not met by 2 of 5 readers, however, largely due to false-positive reads based on the misinterpretation of temporal lobe findings. Despite differences in study design, the present findings suggest that automated quantification of tau PET retention in the temporal meta-ROI could prove a suitable measure to support visual reads in clinical practice.

For the data-driven ROIs, overlap in the regions comprising the composite ROIs for both contrasts (i.e., AD dementia vs CU and non-AD) was high but imperfect across tracers. This finding reflects a combination of cohort effects (e.g., the amount and distribution of tau pathology across diagnostic groups within each cohort) and the use of different tracers. While post-mortem studies have shown that [^18^F]flortaucipir, [^18^F]RO948, and [^18^F]MK6240 all bind to AD-type tau tangles [5, 40, 41], these tracers may differ in their sensitivity to other forms of tau pathology such as neuropil threads and dystrophic neurites. Future work using head-to-head study designs will be required to address this.

By applying a linear scaling operation to amyloid PET data, outcome data can be expressed in a common 100-point scale (unit termed “Centiloids”) [24], with zero representing the average value seen in high-certainty Aβ-negative subjects and 100 the average in typical AD patient with mild-to-moderate dementia. Recent work applying this method to multicenter amyloid PET data [42] has shown the Centiloid approach to be feasible and robust; further, by incorporating comparisons with post-mortem measures of Aβ pathology, the authors reported a neuropathology-based Centiloid cut-off for amyloid PET positivity. The present study, while exploratory in nature, provides preliminary support for the development of a Centiloid-like methodology to facilitate comparisons across tau PET tracers. In the interim, our findings indicate that multicohort studies combining different tau tracers are possible if simply classifying particiapnts as tau positive (i.e., temporal meta-ROI > 1.35 SUVR) or negative.

This study has limitations. First, the non-AD groups varied in size and composition. In the TRIAD cohort, for instance, there were no patients with DLB or svPPA. As both diagnoses have been associated with elevated tau PET signal [31, 32, 43–46], this imbalance may explain the somewhat higher AUC values for [^18^F]MK6240 when separating AD dementia from non-AD disorders, in comparison to results using [^18^F]flortaucipir and [^18^F]RO948. The same can be said about the CU individuals scanned with [^18^F]MK6240 as they were on average younger than those for [^18^F]flortaucipir and [^18^F]RO948; this may explain the larger group separation seen with this [^18^F]MK6240. Further studies with [^18^F]MK6240 covering the spectrum around the proposed cut-off are required. The absence of Aβ-positivity in the non-AD group using [^18^F]MK6240 may also explain the finding that SUVR values in the Early tau and temporal meta-ROIs were somewhat higher in the CU group, where 22% were amyloid PET positive [47]. Second, we here focused on differential diagnosis at the dementia stage. Subsequent studies using longitudinal data will be required to examine the role of tau PET in identifying CU and MCI individuals who subsequently progress to AD dementia. Though cohort differences precluded cross-cohort evaluations, the present study was not intended as a head-to-head comparison of the three tracers for diagnostic purposes. Third, these results may not generalize to other new tau PET tracers currently entering the field such as [^18^F]PI-2620, [^18^F]GTP1, or [^18^F]JNJ-067 [4]. Lastly, though not the aim of the present study, these findings cannot be extrapolated with respect to what ROI(s)/cut-off(s) may prove best for longitudinal applications (e.g., predicting cognitive decline or tau accumulation). In addition to the large sample size, strengths of this study include the incorporation of both CU and non-AD subjects as diagnostic groups and the use of three different tau PET tracers. 

## Conclusion

A common-ROI encompassing parts of the temporal lobe (i.e., Braak I/IV) can be used for differential diagnosis of dementia patients with [^18^F]flortaucipir, [^18^F]RO948, and [^18^F]MK6240 tau PET, and that using very similar cut-offs of around 1.35 SUVR. These findings support the concept of a common scale for tau PET.

## Supplementary information

ESM 1 (DOCX 1.59 mb)
