# Supplementary material for: A multicenter comparison of [18F]flortaucipir, [18F]RO948, and [18F]MK6240 tau PET tracers to detect a common target ROI for differential diagnosis
Source: Eur J Nucl Med Mol Imaging. 2021 May 27;48(7):2295–305. doi: 10.1007/s00259-021-05401-4 (PMC8175317; doi:10.1007/s00259-021-05401-4)
Supplement: Supplementary file 1 — (DOCX 1.59 mb) [file 259_2021_5401_MOESM1_ESM.docx]

**Supplementary Online Content**

Leuzy A, Pascoal T, Strandberg O, et al. A multi-centre comparison of [^18^F]flortaucipir, [^18^F]RO948 and [^18^F]MK6240 tau PET tracers to detect a common target ROI for differential diagnosis

**Supplementary Table 1.** Cohort details

**Supplementary Table 2.** Inclusion/exclusion criteria for cognitively unimpaired individuals

**Supplementary Table 3.** Diagnostic criteria for Alzheimer’s disease dementia

**Supplementary Table 4.** Diagnostic criteria for non-Alzheimer’s disease dementia disorders

**Supplementary Table 5.** Diagnostic performance (AD dementia vs non-AD) and cut-offs for [^18^F]flortaucipir and [^18^F]RO948 when excluding PD patients without dementia from the non-AD group

**Supplementary Table 6.** Diagnostic performance (AD dementia vs non-AD) and cut-offs for [^18^F]flortaucipir and [^18^F]RO948 when excluding Aβ-positive PD/PDD and DLB patients from the non-AD group

**Supplementary Figure 1.** Dendrograms (data-driven ROI) for the separation of AD dementia and CU individuals using [^18^F]flortaucipir PET

**Supplementary Figure 2.** Dendrograms (data-driven ROI) for the separation of AD dementia and non-AD disorders using [^18^F]flortaucipir PET

**Supplementary Figure 3.** Dendrograms (data-driven ROI) for the separation of AD dementia and CU individuals using [^18^F]RO948 PET

**Supplementary Figure 4.** Dendrograms (data-driven ROI) for the separation of AD dementia and non-AD disorders using [^18^F] RO948 PET

**Supplementary Figure 5.** Dendrograms (data-driven ROI) for the separation of AD dementia and CU individuals using [^18^F] MK6240 PET

**Supplementary Figure 6.** Dendrograms (data-driven ROI) for the separation of AD dementia and non-AD disorders using [^18^F]MK6240 PET

**Supplementary Table 1.** Cohort details

| **[^18^F]Flortaucipir** | |
| --- | --- |
| **Data source** | **Details** |
| Memory Clinic, Skåne University Hospital, Sweden (BioFINDER) | Secondary site |
| Memory Clinic, Ängelholm Hospital, Ängelholm, Sweden (BioFINDER) | Secondary site |
| Memory Disorder Clinic of Gangnam Severance Hospital, Seoul, South Korea | Tertiary site |
| University of California San Francisco Alzheimer Disease Research Center, UCSF, United States | Tertiary site |
| A05E clinical trial, Avid Radiopharmaceuticals | Relationship between [^18^F]flortaucipir and longitudinal cognitive decline was studied using subjects recruited across 25 academic sites (see https://clinicaltrials.gov/ct2/show/NCT02016560 for complete list of sites). |
| Placebo arm of the Expedition-3 study | Effect of passive immunization against Aβ using Solanezumab on disease progression in patients with mild AD dementia (see https://clinicaltrials.gov/ct2/show/NCT02016560 for complete list of sites). |
| **[^18^F]RO948** | |
| **Data source** | **Details** |
| Memory Clinic, Ängelholm Hospital, Ängelholm, Sweden | Secondary site |
| Department of Neurology, Skåne University Hospital, Lund, Sweden | Secondary site |
| **[^18^F]MK6240** | |
| **Data source** | **Details** |
| McGill Centre for Studies in Aging (TRIAD) | Tertiary site |

**Supplementary Table 2.** Inclusion/exclusion criteria for cognitively unimpaired individuals

| For all three tracers, cognitively unimpaired (CU) individuals had no significant neurological or psychiatric illnesses and did not have mil cognitive impairment or dementia [1-3]. They were a mix of research volunteers recruited through advertisements and persons visiting the memory clinic with cognitive complaints but normal performance at neuropsychological testing (i.e., “subjective cognitive decline” [4]). Exclusion criteria included presence of objective cognitive impairment, severe somatic disease, and current alcohol or substance abuse.  **References**  1. Janelidze S, Stomrud E, Palmqvist S, Zetterberg H, van Westen D, Jeromin A, et al. Plasma beta-amyloid in Alzheimer's disease and vascular disease. Sci Rep. 2016;6:26801. doi:10.1038/srep26801.  2. Jack CR, Jr., Bennett DA, Blennow K, Carrillo MC, Dunn B, Haeberlein SB, et al. NIA-AA Research Framework: Toward a biological definition of Alzheimer's disease. Alzheimers Dement. 2018;14:535-62. doi:10.1016/j.jalz.2018.02.018.  3. Mattsson N, Insel PS, Palmqvist S, Stomrud E, van Westen D, Minthon L, et al. Increased amyloidogenic APP processing in APOE varepsilon4-negative individuals with cerebral beta-amyloidosis. Nat Commun. 2016;7:10918. doi:10.1038/ncomms10918.  4. Jessen F, Amariglio RE, van Boxtel M, Breteler M, Ceccaldi M, Chetelat G, et al. A conceptual framework for research on subjective cognitive decline in preclinical Alzheimer's disease. Alzheimers Dement. 2014;10:844-52. doi:10.1016/j.jalz.2014.01.001. |
| --- |

**Supplementary Table 3.** Diagnostic criteria for Alzheimer’s disease dementia

| Alzheimer’s disease dementia patients met the National Institute on Aging and the Alzheimer’s Association criteria for probable Alzheimer’s disease, as determined by a physician [1].  **References**  1. McKhann G, Drachman D, Folstein M, Katzman R, Price D, Stadlan EM. Clinical diagnosis of Alzheimer's disease: report of the NINCDS-ADRDA Work Group under the auspices of Department of Health and Human Services Task Force on Alzheimer's Disease. Neurology. 1984;34:939-44. doi:10.1212/wnl.34.7.939. |
| --- |

**Supplementary Table 4.** Diagnostic criteria for non-Alzheimer’s disease dementia disorders

| **Diagnosis** | **Criteria** | **Reference** |
| --- | --- | --- |
| Corticobasal syndrome | Armstrong | [1] |
| Non-fluent variant PPA | Gorno-Tempini | [2] |
| Semantic variant PPA | Gorno-Tempini | [2] |
| Progressive supranuclear palsy | PSP study group | [3] |
| Dementia with Lewy bodies | DLB consortium | [4] |
| Parkinson disease | New International PD and MD Society criteria | [5] |
| Behavioral variant FTD | FTDC | [6] |
| Multiple system atrophy | Gilman | [7] |

NIA-AA = National Institute on Aging and Alzheimer’s Association workgroup; FTDC = International Behavioural Variant FTD Criteria Consortium; PD = Parkinson’s disease; MD = Movement disorders

**References**

1. Armstrong MJ, Litvan I, Lang AE, Bak TH, Bhatia KP, Borroni B, et al. Criteria for the diagnosis of corticobasal degeneration. Neurology. 2013;80:496-503. doi:10.1212/WNL.0b013e31827f0fd1.

2. Gorno-Tempini ML, Hillis AE, Weintraub S, Kertesz A, Mendez M, Cappa SF, et al. Classification of primary progressive aphasia and its variants. Neurology. 2011;76:1006-14. doi:10.1212/WNL.0b013e31821103e6.

3. Hoglinger GU, Respondek G, Stamelou M, Kurz C, Josephs KA, Lang AE, et al. Clinical diagnosis of progressive supranuclear palsy: The movement disorder society criteria. Mov Disord. 2017;32:853-64. doi:10.1002/mds.26987.

4. McKeith IG, Boeve BF, Dickson DW, Halliday G, Taylor JP, Weintraub D, et al. Diagnosis and management of dementia with Lewy bodies: Fourth consensus report of the DLB Consortium. Neurology. 2017;89:88-100. doi:10.1212/WNL.0000000000004058.

5. Postuma RB, Berg D, Adler CH, Bloem BR, Chan P, Deuschl G, et al. The new definition and diagnostic criteria of Parkinson's disease. Lancet Neurol. 2016;15:546-8. doi:10.1016/S1474-4422(16)00116-2.

6. Rascovsky K, Hodges JR, Knopman D, Mendez MF, Kramer JH, Neuhaus J, et al. Sensitivity of revised diagnostic criteria for the behavioural variant of frontotemporal dementia. Brain. 2011;134:2456-77. doi:10.1093/brain/awr179.

7. Gilman S, Wenning GK, Low PA, Brooks DJ, Mathias CJ, Trojanowski JQ, et al. Second consensus statement on the diagnosis of multiple system atrophy. Neurology. 2008;71:670-6. doi:10.1212/01.wnl.0000324625.00404.15.

**Supplementary Table 5.** Diagnostic performance (AD dementia vs non-AD) and cut-offs for [^18^F]flortaucipir and [^18^F]RO948 when excluding PD patients without dementia from the non-AD group

|  | AUC (95% CI) | Cut–point (95% CI) | Sensitivity (95% CI) | Specificity (95% CI) |
| --- | --- | --- | --- | --- |
| **[^18^F]Flortaucipir** | | | | |
| ***AD vs Non–AD*** |  |  |  |  |
| Entorhinal cortex | 0.895 (0.856-0.933) | 1.37 (1.26, 1.48) | 78.86 (71.54, 86.18) | 86.30 (80.62, 91.25) |
| Early tau | 0.907 (0.873-0.945) | 1.31 (1.17, 1.36) | 86.18 (79.67, 92.68) | 88.12 (82.50, 93.12) |
| Temporal meta-ROI | 0.910 (0.873-0.946) | 1.36 (1.29, 1.44) | 87.80 (82.11, 93.50) | 85.62 (0.80, 90.62) |
| Neocortical meta-ROI | 0.846 (0.799-0.892) | 1.19 (1.09, 1.23) | 82.11 (74.80, 88.62) | 78.12 (71.25, 84.38) |
| Data–driven^1^ | 0.904 (0.867-0.942) | 1.32 (1.28, 1.39) | 86.20 (80.49, 91.87) | 83.75 (77.50, 89.38) |
| **[^18^F]RO948** | | | | |
| ***AD vs Non–AD*** |  |  |  |  |
| Entorhinal cortex | 0.937 (0.902-0.971) | 1.47 (1.44, 1.70) | 83.30 (75.49, 90.20) | 92.97 (88.73, 97.20) |
| Early tau | 0.939 (0.911-0.971) | 1.35 (1.28, 1.42) | 83.33 (76.47, 90.20) | 92.25 (87.32, 96.48) |
| Temporal Meta-ROI | 0.941 (0.910-0.971) | 1.36 (1.21, 1.43) | 85.29 (77.45, 91.18) | 89.44 (83.80, 94.37) |
| Neocortical meta-ROI | 0.879 (0.836-0.924) | 1.13 (1.03, 1.18) | 75.49 (66.67, 83.33) | 85.92 (80.26, 91.55) |
| Data–driven^2^ | 0.937 (0.906-0.974) | 1.43 (1.38, 1.58) | 90.20 (84.31, 95.10) | 89.44 (83.80, 94.37) |

^1^ Entorhinal cortex, amygdala, parahippocampus and inferior temporal; ^2^ Entorhinal cortex, parahippocampus, amygdala, fusiform gyrus and inferior temporal cortex

**Supplementary Table 6.** Diagnostic performance (AD dementia vs non-AD) and cut-offs for [^18^F]flortaucipir and [^18^F]RO948 when excluding Aβ-positive PD/PDD and DLB patients from the non-AD group

|  | AUC (95% CI) | Cut–point (95% CI) | Sensitivity (95% CI) | Specificity (95% CI) |
| --- | --- | --- | --- | --- |
| **[^18^F]Flortaucipir** | | | | |
| ***AD vs Non–AD*** |  |  |  |  |
| Entorhinal cortex | 0.927 (0.895-0.959) | 1.37 (1.26, 1.48) | 0.870 (0.806-0.935) | 0.863 (0.806-0.919) |
| Early tau | 0.934 (0.904-0.965) | 1.31 (1.17, 1.36) | 0.925 (0.870-0.972) | 0.881 (0.831-0.932) |
| Temporal meta-ROI | 0.935 (0.904-0.965) | 1.36 (1.29, 1.44) | 0.944 (0.898-0.982) | 0.856 (0.800-0.906) |
| Neocortical meta-ROI | 0.873 (0.831-0.916) | 1.19 (1.09, 1.23) | 0.879 (0.814-0.935) | 0.782 (0.713-0.844) |
| Data–driven^1^ | 0.932 (0.901-0.963) | 1.32 (1.28, 1.39) | 0.926 (0.870-0.972) | 0.838 (0.781-0.893) |
| **[^18^F]RO948** | | | | |
| ***AD vs Non–AD*** |  |  |  |  |
| Entorhinal cortex | 0.970 (0.942-0.998) | 1.47 (1.44, 1.70) | 0.929 (0.869-0.976) | 0.930 (0.887-0.972) |
| Early tau | 0.962 (0.936-0.962) | 1.35 (1.28, 1.42) | 0.905 (0.833-0.964) | 0.922 (0.873-0.965) |
| Temporal Meta-ROI | 0.960 (0.933-0.987) | 1.36 (1.21, 1.43) | 0.894 (0.845-0.944) | 0.917 (0.845-0.965) |
| Neocortical meta-ROI | 0.893 (0.850-0.935) | 1.13 (1.03, 1.18) | 0.774 (0.679-0.857) | 0.859 (0.803-0.916) |
| Data–driven^2^ | 0.968 (0.937-0.998) | 1.43 (1.38, 1.58) | 0.964 (0.929-1) | 0.894 (0.845-0.944) |

^1^ Entorhinal cortex, amygdala, parahippocampus and inferior temporal; ^2^ Entorhinal cortex, parahippocampus, amygdala, fusiform gyrus and inferior temporal cortex

**Supplementary Figure 1.** Dendrogram (data-driven ROI) for the separation of AD dementia from cognitively unimpaired individuals using [^18^F]flortaucipir PET

**
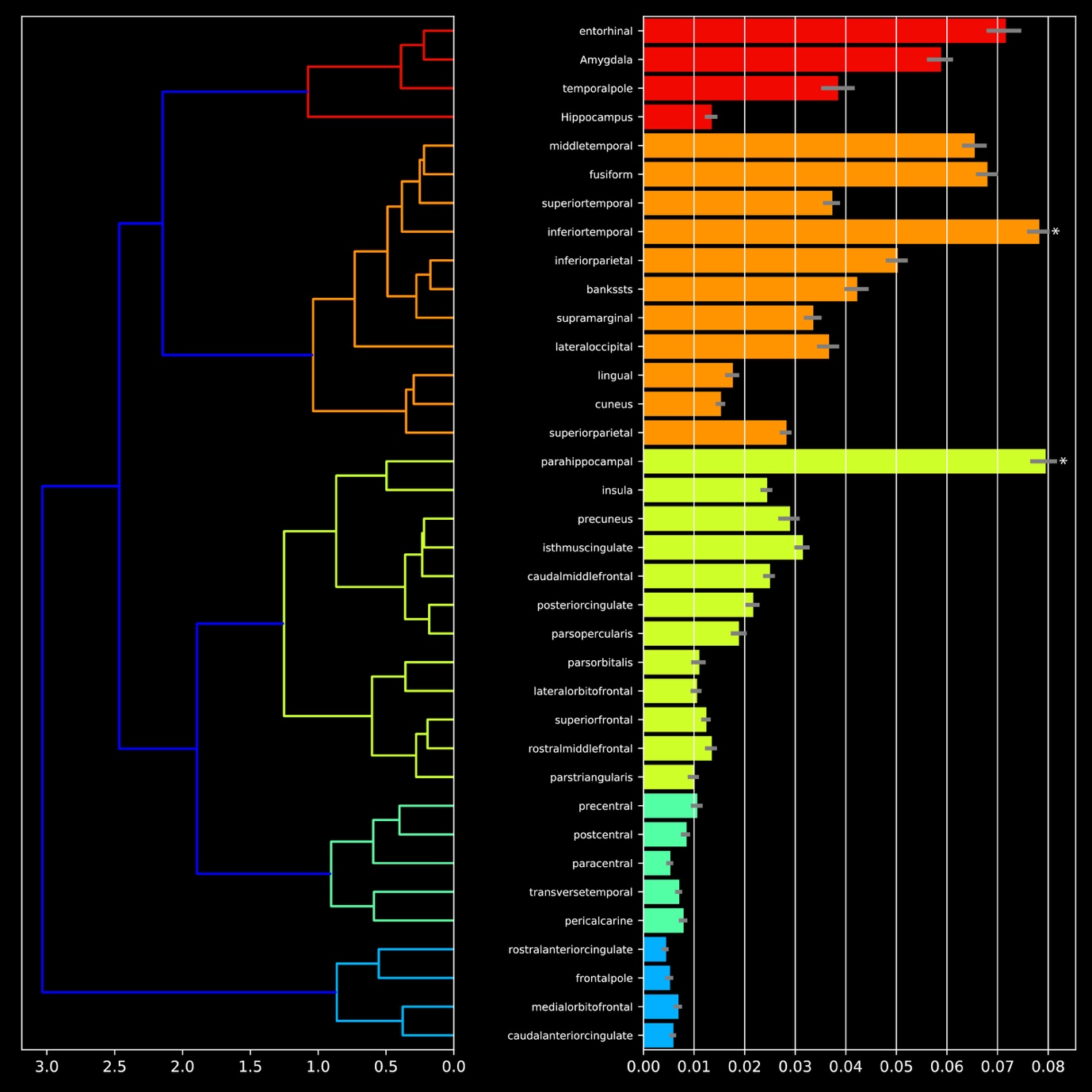
**

To deal with multicollinearity, hierarchical clustering of all variables Spearman’s correlation matrix and resulting dendrogram has been used to group the feature importance scores from the Extra Trees algorithm for the separation of Alzheimer’s disease (AD) dementia patients from cognitively unimpaired (CU) individuals using [^18^F]flortaucipir SUVR data. FreeSurfer regions are shown along the vertical axis with feature importance on the horizontal axis (higher score indicating greater importance for group separation). White stars indicate the FreeSurfer regions that, when combined (i.e., into data-driven ROI), provided the best separation between AD dementia patients and CU individuals in terms of area under the receiver operating characteristic curve. Cluster colors corresponds to an arbitrary, but constant, cluster distance threshold of 1.5 (leftward x-axis). All separable subclusters below this threshold have a smaller pairwise intercluster distance. The constant threshold will result in a different number of colors in each dendrogram, which is meant to reflect the relative separability into groups of lowest possible variance. The arbitrary distance threshold was chosen to give a good visual overview of the cluster separability and allows direct comparison of resulting group numbers between different dendrograms.

**Supplementary Figure 2.** Dendrogram (data-driven ROI) for the separation of AD dementia from non-AD disorders using [^18^F]flortaucipir PET


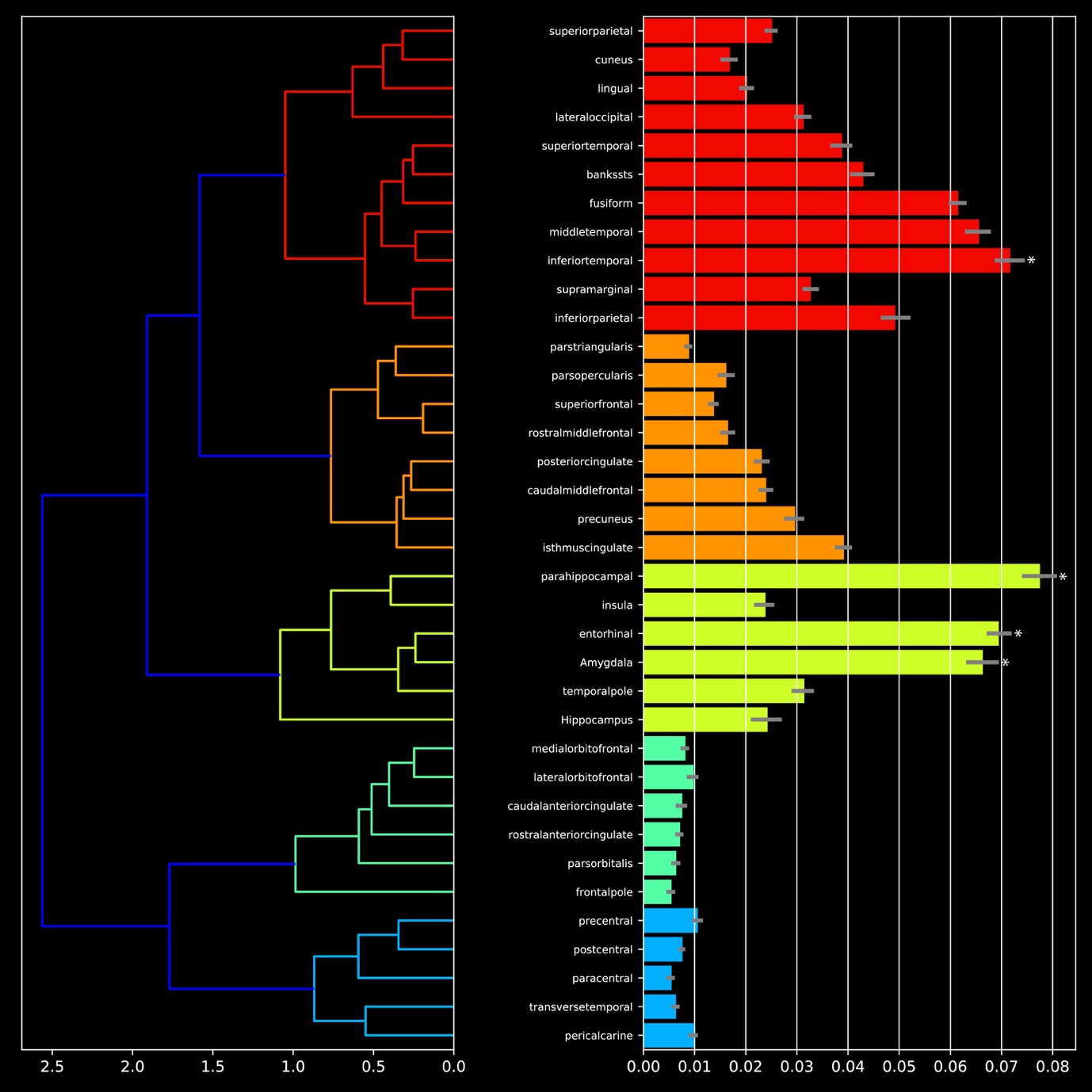


To deal with multicollinearity, hierarchical clustering of all variables Spearman’s correlation matrix and resulting dendrogram has been used to group the feature importance scores from the Extra Trees algorithm for the separation of Alzheimer’s disease (AD) dementia patients from non-AD dementia disorders using [^18^F]flortaucipir SUVR data. FreeSurfer regions are shown along the vertical axis with feature importance on the horizontal axis (higher score indicating greater importance for group separation). White stars indicate the FreeSurfer regions that, when combined (i.e., into data-driven ROI), provided the best separation between AD dementia patients and non-AD dementia disorders in terms of area under the receiver operating characteristic curve. Cluster colors corresponds to an arbitrary, but constant, cluster distance threshold of 1.5 (leftward x-axis). All separable subclusters below this threshold have a smaller pairwise intercluster distance. The constant threshold will result in a different number of colors in each dendrogram, which is meant to reflect the relative separability into groups of lowest possible variance. The arbitrary distance threshold was chosen to give a good visual overview of the cluster separability and allows direct comparison of resulting group numbers between different dendrograms.

**Supplementary Figure 3.** Dendrogram (data-driven ROI) for the separation of AD dementia from cognitively unimpaired individuals using [^18^F]RO948

**
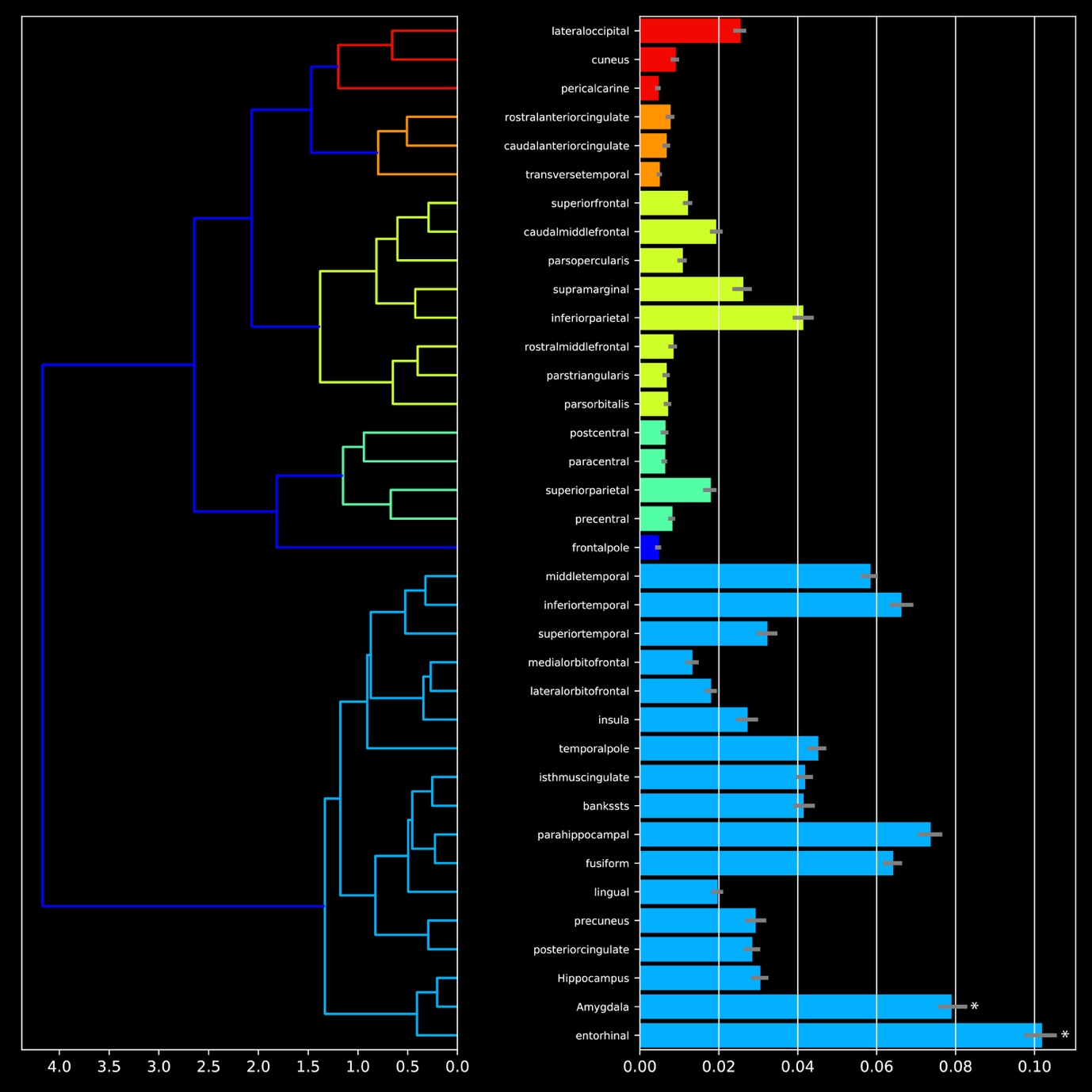
**

To deal with multicollinearity, hierarchical clustering of all variables Spearman’s correlation matrix and resulting dendrogram has been used to group the feature importance scores from the Extra Trees algorithm for the separation of Alzheimer’s disease (AD) dementia patients from cognitively unimpaired (CU) individuals using [^18^F]RO948 SUVR data. FreeSurfer regions are shown along the vertical axis with feature importance on the horizontal axis (higher score indicating greater importance for group separation). White stars indicate the FreeSurfer regions that, when combined (i.e., into data-driven ROI), provided the best separation between AD dementia patients and CU individuals in terms of area under the receiver operating characteristic curve. Cluster colors corresponds to an arbitrary, but constant, cluster distance threshold of 1.5 (leftward x-axis). All separable subclusters below this threshold have a smaller pairwise intercluster distance. The constant threshold will result in a different number of colors in each dendrogram, which is meant to reflect the relative separability into groups of lowest possible variance. The arbitrary distance threshold was chosen to give a good visual overview of the cluster separability and allows direct comparison of resulting group numbers between different dendrograms.

**Supplementary Figure 4.** Dendrogram (data-driven ROI) for the separation of AD dementia from non-AD disorders using [^18^F]RO948 PET

**
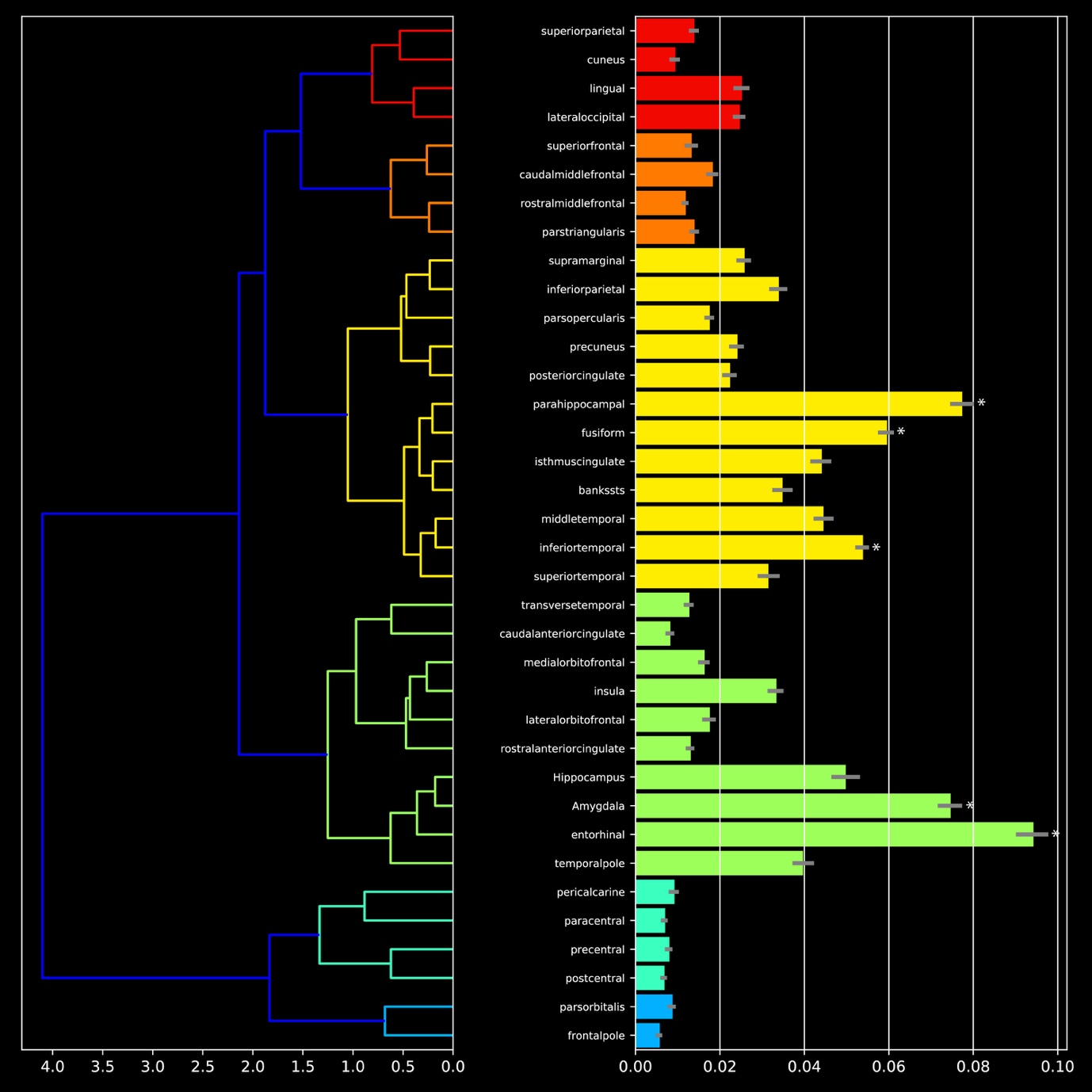
**

To deal with multicollinearity, hierarchical clustering of all variables Spearman’s correlation matrix and resulting dendrogram has been used to group the feature importance scores from the Extra Trees algorithm for the separation of Alzheimer’s disease (AD) dementia patients from non-AD dementia disorders using [^18^F]RO948 SUVR data. FreeSurfer regions are shown along the vertical axis with feature importance on the horizontal axis (higher score indicating greater importance for group separation). White stars indicate the FreeSurfer regions that, when combined (i.e., into data-driven ROI), provided the best separation between AD dementia patients and non-AD dementia disorders in terms of area under the receiver operating characteristic curve. Cluster colors corresponds to an arbitrary, but constant, cluster distance threshold of 1.5 (leftward x-axis). All separable subclusters below this threshold have a smaller pairwise intercluster distance. The constant threshold will result in a different number of colors in each dendrogram, which is meant to reflect the relative separability into groups of lowest possible variance. The arbitrary distance threshold was chosen to give a good visual overview of the cluster separability and allows direct comparison of resulting group numbers between different dendrograms.

**Supplementary Figure 5.** Dendrogram (data-driven ROI) for the separation of AD dementia from cognitively unimpaired individuals using [^18^F]MK6240 PET

**
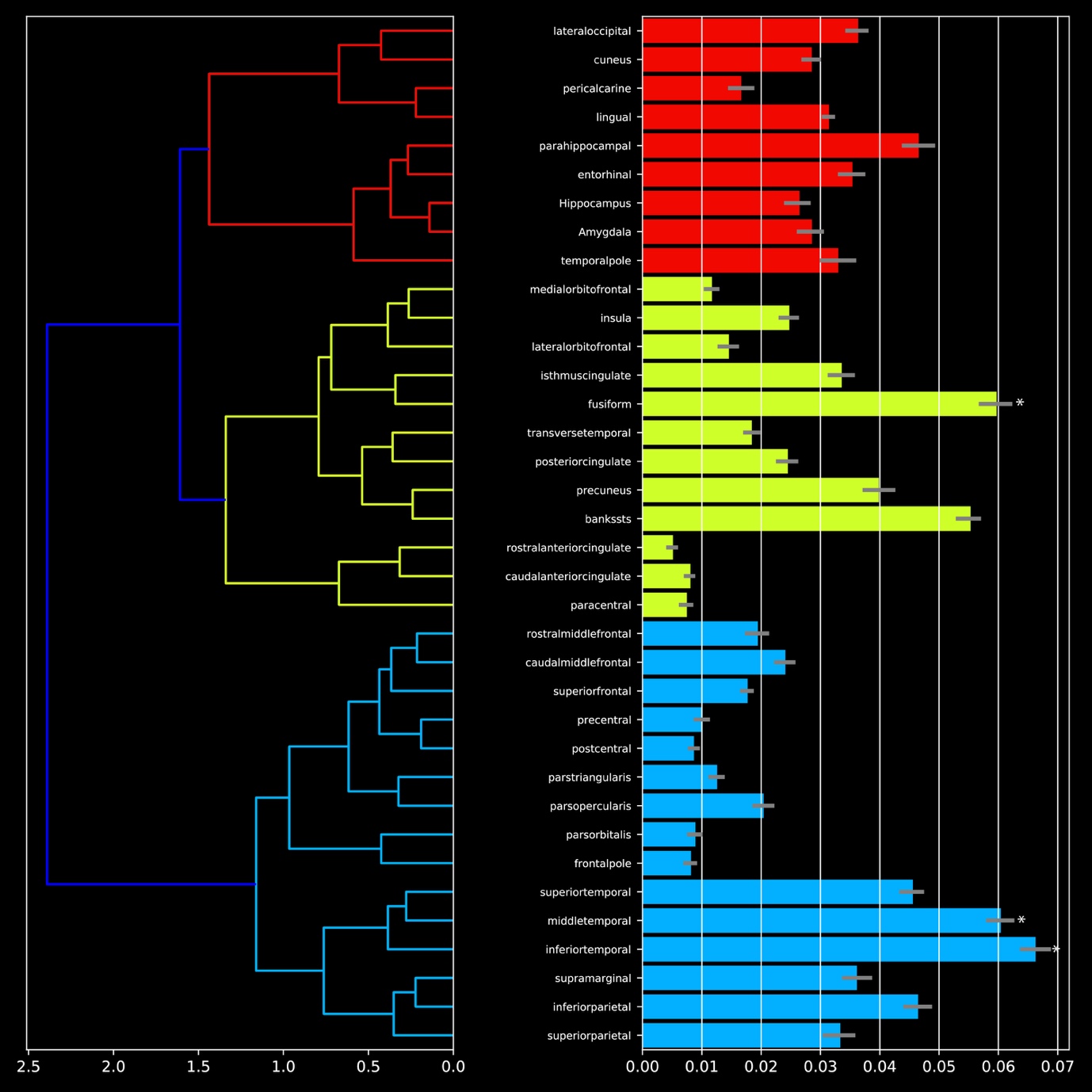
**

To deal with multicollinearity, hierarchical clustering of all variables Spearman’s correlation matrix and resulting dendrogram has been used to group the feature importance scores from the Extra Trees algorithm for the separation of Alzheimer’s disease (AD) dementia patients from cognitively unimpaired (CU) individuals using [^18^F]MK6240 SUVR data. FreeSurfer regions are shown along the vertical axis with feature importance on the horizontal axis (higher score indicating greater importance for group separation). White stars indicate the FreeSurfer regions that, when combined (i.e., into data-driven ROI), provided the best separation between AD dementia patients and CU individuals in terms of area under the receiver operating characteristic curve. Cluster colors corresponds to an arbitrary, but constant, cluster distance threshold of 1.5 (leftward x-axis). All separable subclusters below this threshold have a smaller pairwise intercluster distance. The constant threshold will result in a different number of colors in each dendrogram, which is meant to reflect the relative separability into groups of lowest possible variance. The arbitrary distance threshold was chosen to give a good visual overview of the cluster separability and allows direct comparison of resulting group numbers between different dendrograms.

**Supplementary Figure 6.** Dendrogram (data-driven ROI) for the separation of AD dementia from non-AD disorders using [^18^F]MK6240 PET


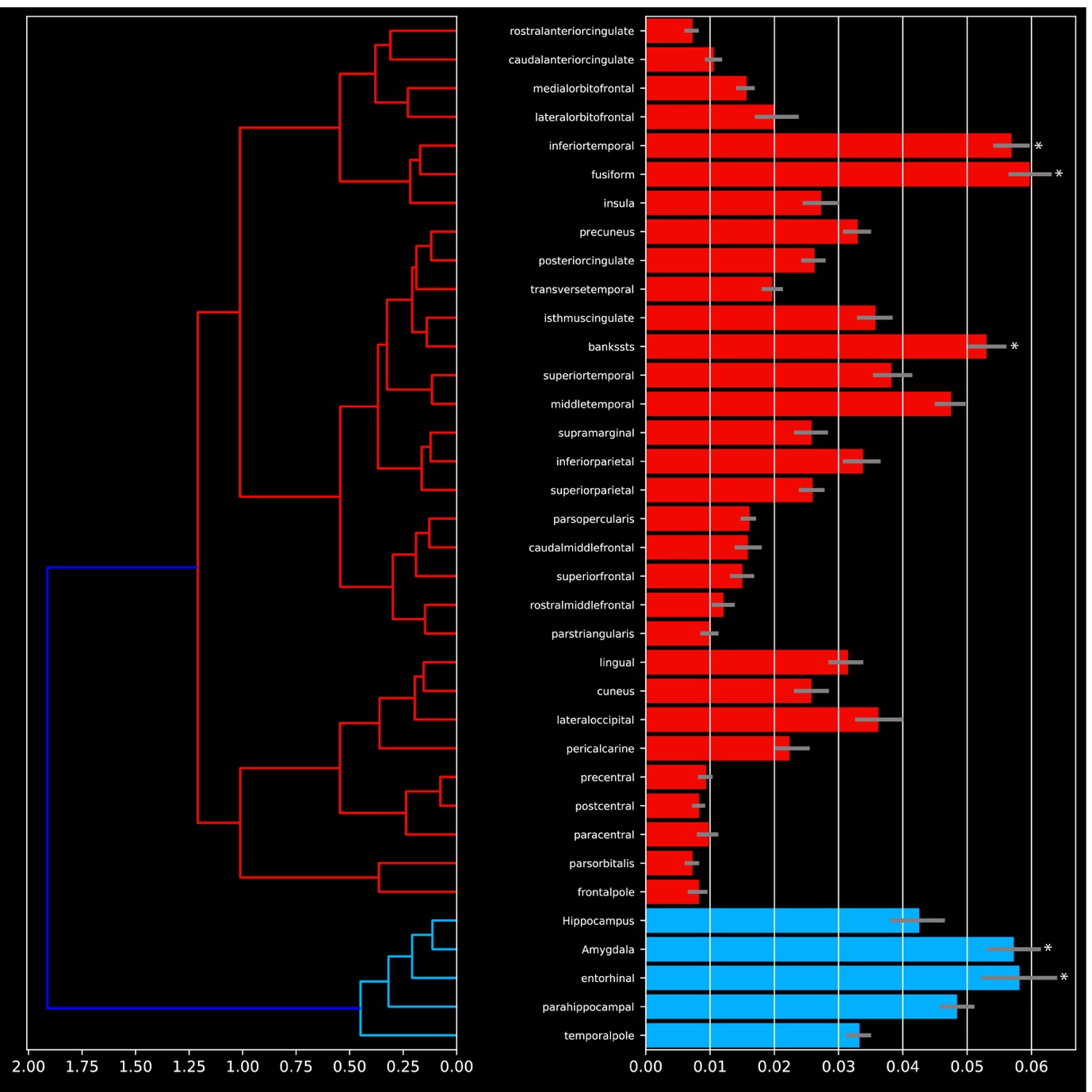


To deal with multicollinearity, hierarchical clustering of all variables Spearman’s correlation matrix and resulting dendrogram has been used to group the feature importance scores from the Extra Trees algorithm for the separation of Alzheimer’s disease (AD) dementia patients from non-AD dementia disorders using [^18^F]MK6240 SUVR data. FreeSurfer regions are shown along the vertical axis with feature importance on the horizontal axis (higher score indicating greater importance for group separation). White stars indicate the FreeSurfer regions that, when combined (i.e., into data-driven ROI), provided the best separation between AD dementia patients and non-AD dementia disorders in terms of area under the receiver operating characteristic curve. Cluster colors corresponds to an arbitrary, but constant, cluster distance threshold of 1.5 (leftward x-axis). All separable subclusters below this threshold have a smaller pairwise intercluster distance. The constant threshold will result in a different number of colors in each dendrogram, which is meant to reflect the relative separability into groups of lowest possible variance. The arbitrary distance threshold was chosen to give a good visual overview of the cluster separability and allows direct comparison of resulting group numbers between different dendrograms.

**Supplementary Table 4.** Summary of diagnostic performance (AD dementia vs non-AD disorders, excluding Parkinson’s disease without

dementia) and cut-offs for [^18^F]flortaucipir and [^18^F]RO948

|  | AUC (95% CI) | Cut–point (95% CI) | Sensitivity (95% CI) | Specificity (95% CI) |
| --- | --- | --- | --- | --- |
| **[^18^F]Flortaucipir** | | | | |
| ***AD vs Non–AD^*^*** |  |  |  |  |
| Entorhinal cortex | 0.895 (0.856-0.933) | 1.37 (1.26, 1.48) | 78.86 (71.54, 86.18) | 86.30 (80.62, 91.25) |
| Early tau | 0.907 (0.873-0.945) | 1.31 (1.17, 1.36) | 86.18 (79.67, 92.68) | 88.12 (82.50, 93.12) |
| Temporal meta-ROI | 0.910 (0.873-0.946) | 1.36 (1.29, 1.44) | 87.80 (82.11, 93.50) | 85.62 (0.80, 90.62) |
| Neocortical meta-ROI | 0.846 (0.799-0.892) | 1.19 (1.09, 1.23) | 82.11 (74.80, 88.62) | 78.12 (71.25, 84.38) |
| Data–driven^1^ | 0.904 (0.867-0.942) | 1.32 (1.28, 1.39) | 86.20 (80.49, 91.87) | 83.75 (77.50, 89.38) |
| **[^18^F]RO948** | | | | |
| ***AD vs Non–AD^**^*** |  |  |  |  |
| Entorhinal cortex | 0.937 (0.902-0.971) | 1.47 (1.44, 1.70) | 83.30 (75.49, 90.20) | 92.97 (88.73, 97.20) |
| Early tau | 0.939 (0.911-0.971) | 1.35 (1.28, 1.42) | 83.33 (76.47, 90.20) | 92.25 (87.32, 96.48) |
| Temporal Meta-ROI | 0.941 (0.910-0.971) | 1.36 (1.21, 1.43) | 85.29 (77.45, 91.18) | 89.44 (83.80, 94.37) |
| Neocortical meta-ROI | 0.879 (0.836-0.924) | 1.13 (1.03, 1.18) | 75.49 (66.67, 83.33) | 85.92 (80.26, 91.55) |
| Data–driven^2^ | 0.937 (0.906-0.974) | 1.43 (1.38, 1.58) | 90.20 (84.31, 95.10) | 89.44 (83.80, 94.37) |

^*^ 55 Parkinson’s disease patients without dementia were excluded from the non-AD group; ^**^ 41 Parkinson’s disease patients without dementia were excluded from the non-AD group. Similar to the main analyses, no significant differences between area under the receiver operating characteristic curve (AUC) values were seen between the best performing theory driven ROI and the data-driven ROIs. Further, 1.36 SUVR was identified as the cut-point for the temporal meta-ROI. ^1^ Entorhinal cortex, amygdala, parahippocampus and inferior temporal cortex; ^2^ Entorhinal cortex, parahippocampus, amygdala, fusiform gyrus and inferior temporal cortex
